# Understanding childbirth practices as an organizational cultural phenomenon: a conceptual framework

**DOI:** 10.1186/1471-2393-13-205

**Published:** 2013-11-11

**Authors:** Roxana Behruzi, Marie Hatem, Lise Goulet, William Fraser, Chizuru Misago

**Affiliations:** 1 Department of Family Medicine, McGill University, Faculty of Medicine, Montreal, Canada; 2 Department of Social and Preventive Medicine, Université de Montréal, Faculty of Medicine, Montreal, Canada; 3 Department of Obstetrics and Gynecology, Université de Montréal, Faculty of Medicine, Montreal, Canada; 4 Department of International and Cultural Study, Tsuda College, Kodaira, Japan

**Keywords:** Humanization of childbirth, Organizational culture, Social birth, Conceptual framework

## Abstract

Understanding the main values and beliefs that might promote humanized birth practices in the specialized hospitals requires articulating the theoretical knowledge of the social and cultural characteristics of the childbirth field and the relations between these and the institution. This paper aims to provide a conceptual framework allowing examination of childbirth practices through the lens of an organizational culture theory. A literature review performed to extrapolate the social and cultural factors contribute to birth practices and the factors likely overlap and mutually reinforce one another, instead of complying with the organizational culture of the birth place. The proposed conceptual framework in this paper examined childbirth patterns as an organizational cultural phenomenon in a highly specialized hospital, in Montreal, Canada. Allaire and Firsirotu’s organizational culture theory served as a guide in the development of the framework. We discussed the application of our conceptual model in understanding the influences of organizational culture components in the humanization of birth practices in the highly specialized hospitals and explained how these components configure both the birth practice and women’s choice in highly specialized hospitals. The proposed framework can be used as a tool for understanding the barriers and facilitating factors encountered birth practices in specialized hospitals.

## Introduction

The use of medical interventions such as epidural analgesia, electronic fetal monitoring (EFM), and induction of labor has dramatically increased in recent years in Canada. The results of a recent Canadian study showed that 57.3% of women received epidural analgesia, about 90.8% EFM, and 44% induction of labor [1,2]. The total C-section rate in Canada in 2005–06 was 26.3%, and this rate was 81.9% among women who had a previous C-section [3]. The previous research on medicalization of birth, as well as routine obstetric interventions such a using epidural analgesia report a need for further research for possible severe adverse events, and increased rate of intervention [4-6].

The humanization of childbirth is arguably an alternative model to the medical and technological models. Most of the previous literature defines the humanization of birth as birth without any unnecessary medical intervention. A women-centered care approach in which women are respected regarding their values, beliefs, autonomy, choices, and their control over their bodies and births are considered as key concepts of humanized birth care [7-10].

Humanized care is a changing and developing process. Humanized birth care in high-risk pregnancies aims at enhancing patient care for the improvement of the birthing experience in hospitals [11]. In a highly specialized hospital, many of the patients are high-risk, so they need specific attention and care. Some high-risk ante partum patients and their families have to adapt to long hospital stay and confinement to bed. On the other hand, labeling women as a high obstetric risk produces stress and anxiety which can influence the outcome of pregnancy [12]. Previous research has shown that a hospital’s policies and procedures, inadequate staffing, technology-focused care, and a lack of continuity of care are barriers to a more humanized birth approach in specialized hospitals [13]. Lack of continuity of care is an important barrier to humanized birth care in almost all hospital settings in Canada. A survey by the Public Health Agency of the Canadian Perinatal Surveillance System (CPSS) showed that 50.6% of women reported that they did not have the same caregiver both prenatally and at birth [1].

Most of the existing studies on childbirth still do not manage to stress the importance of the embedded social and cultural norms of an institution or their consequences on birth practices. Moreover, a theoretical framework for studying these issues from an organizational/cultural perspective has not yet been developed. This paper aims to provide a conceptual framework allowing examination of childbirth practices in specialized hospitals through the lens of organizational culture.

We start with a discussion about the social and cultural aspects of childbirth. The feminist framework of childbirth is discussed, since this is arguably the most important scholarly model of birth that supports a humanized birth approach. We will further discuss the limits of feminist literature regarding the analysis of medicalized birth practices and characterize childbirth as an embedded organizational culture event, proposing to study it from an organizational culture perspective. Using Allaire and Firsirotu’s (1984) theory of organizational culture, we will finally propose our conceptual framework and show its application in a highly specialized hospital.

### Birth as a social and cultural phenomenon

Childbirth has both a biological and a cultural definition. It is also a political and social phenomenon [14]. Esposito (1999) argued that social and cultural power is what creates the potential for diversity in birth, beliefs, practices, and experiences. Liamputtong stated that “the social meaning of birth is shaped by the society in which the birthing women live” [15]. Feminist researchers have also argued that our cultural attitudes towards birth differ according to the individuals’ social culture, social class, and social resources [15-17]. For example, middle-class women seek more medical technology as a way to control their births [15]. According to Davis-Floyd, humans’ actions such as the cultural creation of traditions, customs, and rules construct childbirth practices directly. These actions take place through social interactions, communication, and exchanges inside the social institutions [18].

Considering pregnancy as a socially constructed event, Schneider (2002) assumed that “women’s views reflect, more or less, the views of the health professionals, family, friends, and those in the literature” [14]. Klein argued that “women tended to want what the society values and what this technocratic society values is a high technology in almost every aspect of life” [19]. DeVries, in his book *Birth by design*, has emphasized that maternity care systems must be studied in the historical, cultural, and societal settings in which they function. According to DeVries, what women want in birthing shows how women’s desires and needs at birth can both be constructed by and can construct the maternity care they receive [20]. Anderson (2004) also argued that how women view their care and their willingness to receive care during labor and delivery have greatly changed from the 1980s’ notion of having a “natural birth” to an increased request for “medical technology” in the twenty-first century [21].

The social features of birth including cultural ideas and social support systems have an important impact on birth practices. Social scientists have argued that a medicalized birth is determined by embedded cultural ideas in which progress and technological birth practices are defined as a victory of civilized society over the ancient feminine nature of birth. Consequently, women are controlled through more and more medical practices in order to prevent any risk to themselves and their babies [22]. This view of birth helps us understand how birth is perceived and practiced as a socially embedded experience, whilst maintaining an emphasis on the role of hospitals in providing safety.

Social scholars have argued that social dimensions of birth are inherent in natural childbirth whereas a modern-day, escape from society back to nature birth seems impossible because, in real situations, both women and care providers integrate elements of modern medicine into previously natural childbirth [23,24]. Macdonald (2006) stated that the concept of a “natural birth” needed redefinition by the professionals, specifically midwives, politicians, and, of course, women. Macdonald (2006) concluded that the experience of a natural birth in contemporary midwifery in Canada reflects and promotes an understanding of this concept in modern Canadian society. She also makes room for the role of biomedical technology and hospital spaces but supports this through the midwifery logic of caring and choice [23].

The limitation of the existing socio-cultural studies of birth practices is that they fail to explore the organizational culture dimensions of the institution and their role and power over the change in birth practices towards a more humanized one. What kind of socio-cultural opportunities or constraints are imposed on organizations trying to adopt humanized or medicalized birth approaches?

In the following part, we discuss the feminist theory of childbirth as being one of the best frameworks for understanding our proposed conceptual framework. Following this discussion, we highlight our reasons for choosing the organizational culture theory for our conceptual framework.

### The feminist framework of childbirth

Feminist activists have provided a new insight into childbirth and opened the doors to new topics for research including the sociology of childbirth (Rothman, 1982). During the nineteenth and early twentieth centuries the first wave of feminist activists argued persistently for women’s rights to relieve their own suffering, and hence to gain control over the birthing process, the right of extended choices during childbirth, and full control over their body, as well as their reproductive life. The consequences of the struggle of the first wave of feminist activists were beneficial, as women gained the right to use pain relief drugs and to express their preference for or against it; however, women lost control over the process of childbirth, as well as allowing birth to continue to shift from home to hospital [25,26].

In the late 1960s and early 1970s, the second wave of feminist activists began to take an active interest in the “alternative birth” or “natural birth” movement, and once more advocated home birthing as well as midwifery services [27,28]. They became much more aware of how the widespread use of technology caused women problems with their body image and their powerlessness over birth. In this second movement, feminists supported a more humanistic, woman-centered, and holistic approach to pregnancy and childbirth [14,16,29].

Most of the feminist scholars described “natural” or “normal” births as part of a social process which was based on different cultural ideas [22]; however, they ignored the analysis of natural birth from the organizational and cultural perspective.

From the feminist literature it is clear that certain facets leading to the medicalization of birth have developed on a *gender* perspective basis [23]. Some have, for example, criticized men’s control over childbirth. They argue that the establishment of modern medicine and obstetric technology being the cause of changes on women’s normal birth processes to pathological events [17,24,26,30]. Nevertheless, Dillaway and colleagues (2006) criticize the feminist study of birth, and state that previous conceptual approaches that focus solely on gender oppression fail to explain the birthing experience from diverse dimensional standpoints [31]. Although the feminist critiques of medicalized birth care have contributed greatly to our understanding of the patriarchal construction of childbirth as a gendered process, these approaches still rarely consider how these gender issues interact with the “organizational culture” of the birthplace to affect women’s experience.

Moreover, birth practices have been analyzed from a cross-cultural perspective by many anthropologists and feminist scholars [32-34]. The feminist/cultural perspective has contributed to our knowledge of the varieties of birth practices among different cultures [33]. It seems that the medicalized birth system is more embedded in US culture, as US women are less likely to question the use of particular procedures in hospitals [31]. From the feminist cross-cultural studies, we realize how differences between birthplace, race, ethnicity, and the religion of women play a role in their decision-making on medicalized birth. Previous research has shown that most Japanese women prefer to have a natural birth, and avoid epidural analgesia and other medical interventions at birth [35-37]. In contrast, half of Canadian women chose a method of pain relief such as epidural analgesia and 81% rated it as “very helpful” [1]. Davis-Floyd argued that technology is seen as essential in all aspects of US life, and women fully expect a technocratic birth in order to insure that their births are well managed, controlled, and safe. Davis-Floyd’s study showed that 70% of the interviewed women were both excited about and comfortable with their highly technocratic childbirth experience [38].

We also understand that African-American women had more desire for medicalized births because of their historical lack of access to appropriate medical care and mistreatment by professionals [39]. Jewish women, even more than African-American women, embraced the medicalized approach [40].

Finally, the contemporary feminists or “third wave” of feminist activists argue about women’s choice and their positive experience of obstetric technology at birth. The feminist literature has started to uncover women’s own views on medicalized birth, and to show women’s desire for the medical model of birth, such as epidural analgesia, in a hospital setting. The contemporary group of feminists emphasizes that technology is not essentially a male-gendered product for the establishment and continuation of the obstetrician’s authority at birth, and it can serve women’s needs and purposes. Beckett (2005) argues that women can purposefully choose and benefit from the utilization of obstetric technology.

To our knowledge, however, no research has so far looked at how the organizational culture of the hospital setting may change women’s decisions when choosing a specific medical intervention, such as an epidural or a cesarean section. Understanding women’s perceptions of and decisions about medical interventions is only possible if we pay attention to the commonalities and differences in the organizational culture in the environment where birth takes place.

### Choice of the “organizational culture” model theory for childbirth practice

In order to improve childbirth practice, we need to understand the way in which birth is experienced by women and also the “internally consistent and mutually dependent practices and beliefs that exist around it” [33]. Newburn’s (2003) findings demonstrated that women’s needs are not being adequately met in many birth units in hospitals. There was a lack of knowledge among women, particularly those expecting their first baby, about what they should expect from the specific hospital that they chose for their birth setting [41]. The literature shows that the social atmosphere as well as changes in women willingness to accept intervention, greatly influences healthcare professionals’ practice and women’s experience of birth [27,31,37,42,43]. For the majority of women, it is important to have access to epidural analgesia and to a special care unit for the baby [41]. Individual factors, however, such as convenience incentives, the ambient of society and their role in increasing intervention at childbirth have never been addressed through a comprehensive organizational culture model.

Moreover, humanizing childbirth draws away a phenomenon of organizational change. To understand whether hospitals are able to transform themselves, it is not enough to study only their definite rational characteristics. Organizations also include a culture that is formed by values, beliefs, and signification, all of which constitute the foundation of organizational functioning [44]. Study of organizational culture allows us to understand the values and assumptions towards medicalized vs. humanized birth practices in specialized hospitals. Dastmalchian (2000) stated that organizational culture is a unique area in which conceptual work and research can serve as guidance for practitioners [45]. Moreover, Esposito argued that “the process of birth provides a structure around which the social and cultural forces can guide its expressions” [46].

In the present paper, the conceptual model of “organizational culture” introduced by Allaire and Firsirotu (1984) is considered a comprehensive and appropriate theoretical model for the study of childbirth practice in specialized hospitals. This model allows researchers to explore the cultural precipitations of childbirth through the lens of an organizational/ cultural study, in order to understand which childbirth practices work best for which cultures.

Consequently, we will describe the organizational culture theory. After conceptualizing childbirth as an organizational culture phenomenon, we will then introduce the properties of Allaire and Firsirotu’s (1984) organizational culture theory model.

### Definition of organizational culture

Understanding organizational culture is important because culture gives meaning, clarity, and direction to the action of an organization and its members [47]. Organizational culture represents a collective set of expectations, definitions, and memories that characterize how things happen in an organization. Cameron and Schein have defined organizational culture as a pattern of basic assumptions that a group of people has invented, discovered, or developed in learning to cope with problems, such as external adaptation and internal integration [48,49]. According to Deal and Kennedy, a strong culture is a system of informal rules that dictate how people are to behave most of the time, and as such they enable people to feel better about what they do, encouraging them to work harder [50]. Moreover, it seems that the culture influences how people’s perceptions, thoughts, and feelings are related to the length of time they live in this culture and to its age [48]. Understanding the nature of organizational culture is possible by simply observing the group/organization functioning [51].

### Allaire and Firsirotu’s theoretical model of organizational culture

Allaire and Firsirotu (1984) were the first to propose a completely conceptual model of organizational culture, which represents an organization as three inter-related endogenous variables, these being structure, culture, and individuals, all of which are influenced by the external factors surrounding the organization, which in turn include society, history, and contingency [44], and allow researchers to determine the appropriate strategies to improve childbirth practice towards a more humanized and less medical approach.

**Figure 1:**
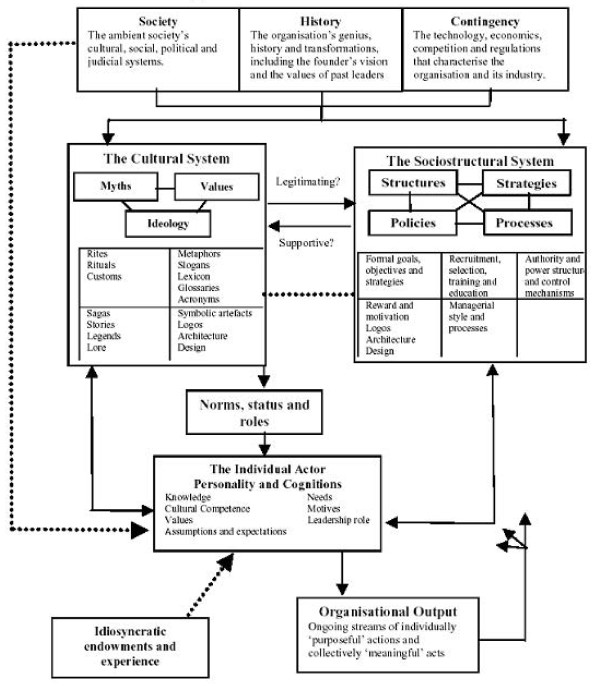
Conceptual framework of organizational culture (Allaire and Firsirotu 1984).

Next, we will cite factors who have contributed to the description of the internal and external components of the organizational culture model theory as explained by Allaire and Firsirotu [44]. We will use these to explain the meaning of this theory (Figure 1).

### External factors

#### Society

The environment in which an organization is constructed, and how this functions, has extensive influence on the organization. Society also defines the judicial and socio-economic context to which an organization must adjust.

#### History

The history of an organization includes how, and why, it has been created. This includes the founder’s vision, the values of past leaders, the successes and failures which the organization has seen, reasons for past leaderships, and finally the routines and rituals that have been exercised over the years. History shapes expectations, past histories of integration, the roots of beliefs, and the expression of the organizational culture, as well as its structural architecture.

#### Contingency

Contingency consists of the technology, economics, competition, and regulations that characterize an organization. The method of functioning and the survival of the organization are deeply adaptive to the type of cultural appearance it portrays and the structural struggles that it may be going through.

### Internal factors

#### Socio-structural factors

These consist of the strategies, structures, policies, and management processes in the organization. They include all aspects of the organization’s functioning, such as formal goals, objectives and strategies, authority, power structure, control mechanisms, rewards and motivation, and managerial processes and style.

#### Cultural factors

Cultural factors manifest themselves strongly in *myths*, *ideologies*, and *values*. This phenomenon is observed in rites and rituals, customs, metaphors, glossaries, lexicons, acronyms, slogans, stories, legends, symbolic artifacts, design, and architecture. The history, the environment, and the contingency of an organization shape culture.

#### Individual factors

These consist of people in different hierarchical levels of leadership roles, as well as passive recipients, who simply contribute to the meaning of the organization. Knowledge, cultural competence, values, assumptions, expectations, needs and motives are the factors which affect the relationships between actors and the extent to which meaning is shared with other actors in the organization.

### Conceptual framework for understanding childbirth practice

**Figure 2:**
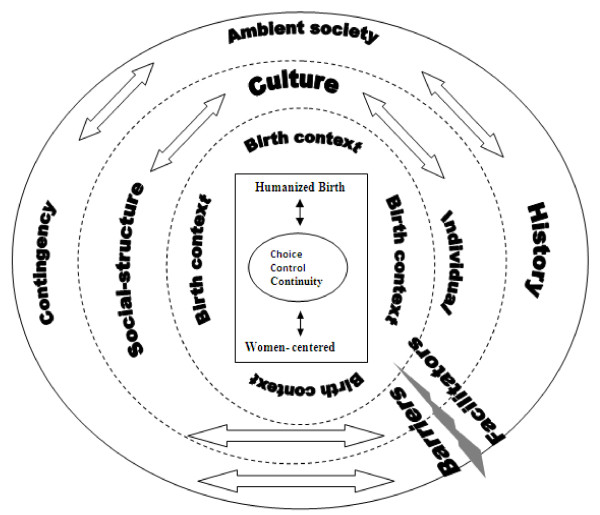
Representation of organizational culture conceptual framework for childbirth practices, adapted from Allaire and Firsirotu organizational culture theory, 1984 and Halabi 2005.

The suggested conceptual framework, adapted from both Allaire and Firsirotu (1984) and Halabi (2005), is presented in (Figure 2). In this framework, a spherical shape reflects the permanence of the relation between the different components pertaining to each of the organization’s levels. The main concept under study, “the humanization of birth” as a potential characteristic of the birth context, has figured in the heart of this organization and has been modeled by it and influenced it in return. This interaction, as well as that lying between Allaire and Firsirotu’s two levels of an organization, is expressed by discontinued lines separating the different spheres of the framework. This represents the permeability between the spheres, which in turn shows that the roles of the different components at the different levels of an organization can be seen as possible facilitators of, or barriers to, the implementation of humanized birth in a specialized hospital. These facilitators and barriers can be raised from the external and internal environment of a highly specialized hospital, and affect humanized birth practice whether independently, or altogether.

The conceptualization of humanized birth in the feminist literature refers to women-centered care, choice, control, and continuity of care [9,16,30,38]. The external sphere represents the exogenous factors of the organization, according to Allaire and Firsirotu (1984): the environment, the history of the organization, and its contingencies. The middle sphere, in turn, represents the endogenous factors of the organization: its structure, its individuals, and its culture.

In order to demonstrate the operationalization of our approach, and in an attempt to reframe these findings using the concepts of our framework, we will adduce some findings taken from the main author’s thesis [52].

### Applying the conceptual framework in practice

By using the proposed framework, the authors of this paper went a step further to examine the proposed theoretical framework in childbirth practices in a highly specialized hospital setting.

A single case study by the main author of this paper for a doctorate thesis carried out in a highly specialized university-affiliated hospital in Montreal, Canada [53]. The study aimed to explore organizational and cultural dimensions that act as barriers or facilitators in the provision of humanized obstetrical care in such a hospital.

The sample consisted of 17 multidisciplinary health professionals and administrators from different hierarchical levels in the hospital, as well as 157 women with different levels of risk, parity, and type of delivery. The data was collected through semi-structured interviews, field notes, documents and archives, the participants’ observations of ten births, and a self-administered questionnaire. The questionnaires were filled out by women during their stay in the postpartum unit. They consisted of 94 questions about the care these women received during their perinatal period. The data collection period spanned the months of November 2007 to March 2008. Both descriptive and qualitative deductive content analyses were performed on the collected data. As a whole, 37% of all pregnancies were diagnosed high-risk; the cesarean section rate was 30% among this sample, and 60% of women who participated in the study received epidural analgesia.

The findings of this study revealed that the participants did not consider the use of technology and medical intervention as opposing the concept of humanized birth care. Most of the women were satisfied with the care they received during their perinatal period in this highly specialized hospital. Women’s satisfaction was observed to be based on the following factors: being in good hands and having a secure and assuring birth, receiving good service, and undergoing a painless childbirth. The women participants’ major cause of satisfaction during childbirth was related to the presence of a competent or specialist professional who could provide a caring and humane manner of assistance during labor and delivery while still applying medical intervention.

The findings of this study showed that both external dimensions of this highly specialized hospital, including history, society, and contingency, and internal dimensions, including culture, structure, and individuals, can affect the humanization of birth care practices, whether they act independently or together.

### External environment of the hospital

The findings of this study also showed that the presence of various organizations and groups within society, such as the “feminist activists”, have had a noticeable influence on childbirth practices in hospitals. This group showed profound support for the rights of women and their families to seek humanized care. The Minister of Health’s new “perinatal” guideline toward de-medicalization of birth in Quebec *(ambient society)* was also an important factor. On the other hand, the stakeholders’ and managers’ aspirations for specialization rather than humanization of care in highly specialized hospitals *(ambient of society)* acts as a major barrier to humanized birth care.

The hospital under study was an integral part of the University Networks Integrated to Health in Montreal and had an objective to improve the quality and continuity of care to mothers and their children, especially after discharge from hospital *(contingency).* The lack of necessary financial support from outside sources was another important *contingency factor*. This served as a *barrier* to this approach. In reality, most money in the hospital was being invested on the physical security of the patient, but investments on the psychological aspects of birth care were not significant. High-risk patients faced with losing a child or a pregnancy, for example, had access to very few psychological resources in this hospital *(contingency).* In certain circumstances, general shortages of staff and the lack of access to resources were the biggest barriers encountered in implementing humanized birth care practice in the studied highly specialized hospital. The stress and anxiety resulting from not having a place of birth, the possibility of not having the choice of a care provider, and the experience of long waiting hours for appointments also led to the dehumanization of birth *(contingency)*.

Analysis of the history of this hospital revealed that the previous and present leaders of the hospital have promoted policies toward humanized birth care, such as accepting companions 24/7, accepting normal pregnancies, providing LDR rooms to all mothers, and integrating midwives into the hospital setting in the near future *(history).* According to the nurses interviewed, these strategies could bring normality to such a specialized environment, as well as ease stress by aiding in the provision of a more humanized form of care for women. On the other hand, becoming a referral center for high-risk pregnancies reinforced the health care provider’s utilization of technical and medical obstetric care and led to the development and implementation of more medicalized, rather than humanized, care in the studied hospital *(history).*

### Internal environment of the hospital

The internal *missions and strategies (structure)* of the studied hospital concentrated on a caring and family-centered approach to childbirth based on the collaboration of family in care. This philosophy and strategy allowed the women’s family to act as partners in care, and this led to a respectful approach toward people and their needs, as well as an environment in which women and their families had the opportunity to grow, learn, and adapt according to their own potential and experiences*.*

The factors related to the *rules and regulations (structure)* of the hospital, such as the hospital’s flexible visiting and companionship rules, were perceived as facilitating factors toward humanized birth. The women participants affirmed that the humanization of birth is more prominent when the staff allows them to have their close relatives nearby, especially during medical interventions or operations. On the contrary, the *rules* regarding discharge in this hospital, which urged mothers to leave the hospital as soon as the discharge was signed, even if they were not psychologically and physically prepared, was seen as a barrier to humanized birth.

A hostel-like service existed in the hospital to accommodate parents for a week after the mother’s discharge without extra charge in case the baby had to stay *(structure).* Having a room in the hostel for mothers who needed to remain somewhere to breastfeed their baby on demand was an important part of the humanization of birth care in the studied hospital. Moreover, the development of the physical environment of the hospital in the near future aimed at providing a friendly and welcoming *physical environment* to the mother and the child while at the same time aiding the implementation of easy access to care and services and the adoption of more space and services for family cohabitation *(structure).* Some women and nurse professionals in this study, however, stated that there were restrictions at the hospital, such as double occupancy of rooms, a lack of space, and a lack of intimacy for families during postpartum *(structure).*

The lack of sufficient communication and teamwork spirit among professionals in this setting was observed to be a barrier in the provision of humanized birth care. Many of the women participants complained about the *professional environment* and lack of communication between health care providers. This caused delays in the transfer of documents and delayed breastfeeding, and sometimes treatment *(structure).* On the other hand, the *training environment* of the hospital and the excessive number of health care professionals interfered with women’s privacy and fostered a lack of intimacy and continuity of care *(structure)*. The interviewed professionals stated that the lack of *human resources*, especially nurses and doctors, made them overflow with work, and that under such conditions it may take longer before they can face the question of the humanization of care *(structure)*.

The professionals and administrators of the hospital expressed *ambitions* for the provision of humanized birth care alongside medical interventional care. From the point of view of the professional participants, medical intervention does not exclude humanized care. These professionals intended to improve satisfaction, safety, assurance, and comfort for the women while providing humanized care *(individual).* The interviewed women were also seen to *value* the medical and technocratic as well as humanized aspects of care *(individual).*

With regard to the *needs and expectations* of the participants*,* most of the women participants said they needed to have the option of a completely pain-free labor and delivery. As a whole, 95 of 157 of the women received epidural analgesia during labor, although most of them had used other methods of relieving pain, such as medication, walking, changing position, breathing, and showering before deciding to have the epidural analgesia. These women felt satisfied with their painless childbirth experience. Noticeably, most of the women participants in the study also perceived that providing epidural analgesia was a humanistic care approach, as it relieved their pain and suffering and allowed them to live through a better child birthing experience *(individual).*

The professionals’ *cultural competency* was another factor that helped the adaptation of multiculturalism and the immigrant population. The professionals stressed the importance of respecting families’ cultural beliefs, desires, needs, preferences, and cultural diversity. Nevertheless, language and communication difficulties between the nurses and some of the parturient women in the postpartum unit were a barrier to the humanization of care; it was undesirable for some women to give birth in a hospital when they did not understand what they were told *(individual)*. Most of the participants also mentioned that their *motivation* was to work for the love of children *(individual)*.

The hospital’s *culture*, such as its festive familial customs and traditions, its ideologies when dealing with patients’ spiritual and religious beliefs, and its valuing of family, largely facilitated the implementation of humanized birth care in this highly specialized hospital. Nevertheless, the institutional *culture* of valuing medical performance was perceived as an obstacle to the humanized birth care approach. Many of the participants argued that both the culture of care around high-risk pregnancies in specialized hospitals and the highly esteemed medical aspects of this care acted as barriers to the humanization of birth *(culture).*

The findings of this study revealed that the birthing environment of the studied hospital contained its own culture and structure as well as its own language and type of technology that mostly focused on risk and its management. In order to explore the facilitating factors and barriers toward the humanization of birth care, one must first redefine the definitions of risk, risk reduction, and risk management within such a setting. The women participants in the studied highly specialized hospital appreciated the technocratic approach to childbirth and even perceived it as a form of humanized birth care, because technology enhanced their feelings of security, assurance, and provided them with a pain-free birth. The establishment and practice of the humanized birth care model in a highly specialized hospital is thus a much greater goal than simplistic opposition to the medicalized birth care model and the technological supremacy associated with it. Empowering women throughout their pregnancy and childbirth, providing psychological supports to high-risk mothers [12], modifying the rules and regulations of institutions to provide more continuity of care, evolving the mechanisms of budget allocation to hospitals, and proposing closer cooperation and better communication between the different professional levels are all important factors that could promote the organization of care with regard to a humanized birth care approach in highly specialized hospitals.

### Implications for future studies

The conceptual framework proposed in this paper can be used as a tool for understanding the barriers and facilitating factors encountered in the humanization of birth practices in specialized hospitals, where a high level of technological and medicalized birth practices exists. The hospital culture, its social context, and its individuals are significant factors involved in the increase in the technocratic model of birth in most modernized and developed countries. The role of contingency factors, such as the rules and regulations, technology, and economic status that impact the specialized hospitals either by promoting or discouraging humanized or medicalized birth approaches, awaits full exploration, which entails the necessity of further investigation in those hospitals. Nevertheless, the conceptual framework is also applicable for examining humanized birth practices in all hospitals regardless of their level of specialty.

## Conclusion

To provide maternity care of optimal quality, public health stakeholders need to be aware of the childbirth practices in different organizations and then insure that these conform to women’s and their families’ needs. The theoretical framework of this study may not be broad enough to allow analysis of all the organizational dimensions and their influences on the provision of optimal care, but at a theoretical and practical level it still has the potential to highlight some components of humanized birth care and the facilitating factors for or barriers to such care in highly specialized hospitals.

## Competing interests

The authors declare that they have no competing interests.

## Authors’ contributions

Four persons have fulfilled the conditions required for authorship. Author 1 (RB) has coordinated the paper from writing its protocol, taking approvals, designing the semi-structured questionnaires, collecting the data, transcriptions, analysis, and redaction of the manuscript. Author 2 (MH) supervised the project from beginning to the end, helped in implementing the research, helped in qualitative analysis and validate the methodology, and participated in drafting the manuscript. Author 3 (LG) also supervised the project, participated in the design of the study and questionnaire development. Authors 4 (WF) and 5 (CM) participated in drafting the manuscript. All authors read and approved the final manuscript.

## Pre-publication history

The pre-publication history for this paper can be accessed here:

http://www.biomedcentral.com/1471-2393/13/205/prepub
